# Association between Vaso-occlusive Crises and Opioid Prescriptions among Patients with Sickle Cell Disease: A Retrospective Claims-based Study

**DOI:** 10.36469/jheor.2020.13348

**Published:** 2020-06-26

**Authors:** Hyeun Ah Kang, Jamie C. Barner, Kristin M. Richards, Menaka Bhor, Jincy Paulose, Abdullah Kutlar

**Affiliations:** 1 The University of Texas at El Paso, School of Pharmacy, El Paso, TX; 2 The University of Texas at Austin, College of Pharmacy, Austin, TX; 3 Novartis Pharmaceuticals Corporation, US Oncology, East Hanover, NJ; 4 Augusta University, Center for Blood Disorders, Augusta, GA

**Keywords:** sickle cell disease, sickle cell, vaso-occlusive crisis, VOC, pain crisis, opioids

## Abstract

**Background/Objectives:**

Among sickle cell disease (SCD) patients, vaso-occlusive crises (VOCs) are recurrent and unpredictable attacks of acute pain. These pain crises are often treated with analgesics, including opioids, which have been associated with misuse and overdose. The aim of this study was to examine the association between VOC events and opioid use and assess the association between opioid prescriptions and health care resource utilization among SCD patients.

**Methods:**

This was a retrospective cohort study using Texas Medicaid medical and prescription claims between September 2011 and August 2016. The index date was the first SCD diagnosis. Patients (2–63 years) with at least one inpatient or two outpatient SCD diagnoses, who were continuously enrolled during 12 months postindex, were included in the study. The primary outcome was number of opioid prescriptions, while the independent variable was number of VOC events. Covariates included age, gender, nonopioid medication use, nonstudy SCD-related medication (penicillin and folic acid) use, evidence of blood transfusions, number of SCD-related complications, number of SCD-related comorbid conditions, and Charlson Comorbidity Index score. Negative binomial regression analysis was used to address study objectives.

**Results:**

Of 3368 included patients, 1978 (58.7%) had at least one opioid prescription with a mean of 4.2 (SD=7.2). Overall, 2071 (61.5%) had at least one VOC event with an average of 2.9 (SD=4.4). The results from the negative binomial regression showed that for every increase in VOC events, the number of opioid prescriptions increased by 9.5% (Incidence rate ratio=1.095, 95% CI: 1.078–1.111; *P* ≤ 0.0001). Other significant covariates associated with higher opioid use included age (13 and older compared to 2–12) and increase in the number of nonopioid pain medications, nonstudy SCD-related medications, and SCD-related complications.

**Conclusions:**

The majority of SCD patients had at least one VOC event and were prescribed opioids during the 12-month study period. We found that each VOC event was associated with a 9.5% increase in the use of opioids. SCD guidelines recommend opioids for the treatment of VOC-related pain. Payers and providers should be aware of opioid use in this population, consider appropriate VOC prevention measures, and provide SCD patients with access to appropriate pain management.

## BACKGROUND

Sickle cell disease (SCD), a rare inherited blood disorder characterized by a defect in the gene for hemoglobin, affects approximately 100 000 people in the United States (US).^1^,^2^ Annual total expenditures for SCD-related emergency department (ED) visits and hospitalizations were estimated from a 2006 nationwide sample to be more than US$2.4 billion overall.^3^ Total SCD-related medical expeditures for children were estimated to be US$335 million in 2005.^4^ The World Health Organization recognizes SCD as a societal health burden^5^ due to the recurrent catastrophic pain events (vasoocclusive crises [VOCs]) and numerous complications (e.g., anemia, life-threatening pneumonia-like illness, acute chest syndrome, cerebrovascular accidents, and splenic and renal dysfunction),^6^–^9^ resulting in costly ED visits and hospitalizations.^10^–^13^ VOCs are the hallmark of SCD and are associated with the majority of SCD patient hospitalizations.^10^ These episodes of excruciating pain can be frequent and unexpected, with most SCD patients experiencing at least one VOC event in their lifetimes.^10^,^14^–^16^ On average, people with SCD have more than six VOC hospitalizations per year, with an average length of stay ranging from 9 to 11 days for severe events.^17^,^18^

Treatment of SCD has focused on preventing VOCs and several treatment options (hydroxyurea, L-glutamine, transfusion) have shown effectiveness in reducing the number of VOCs in clinical trials and/or observational studies.^19^–^21^ One study also revealed that use of hydroxyurea and blood transfusions were associated with lower opioid use.^22^ Hydroxyurea—which was the only US Food and Drug Administration-approved, disease-modifying pharmacologic SCD treatment until 2017—has demonstrated beneficial outcomes for SCD patients for decades.^12^,^19^,^23^–^27^ However, real-world evidence indicates suboptimal adherence,^28^–^30^ resulting in increased health care utilization and costs.^28^ While the goal of treatment is to prevent painful crises, suboptimal adherence and lack of options often result in patients seeking care for pain. Treatment of pain in SCD is important, because if acute pain crises are not treated appropriately, they can develop into chronic pain and possibly evolve into neuropathic pain.^15^,^16^,^31^,^32^ The National Heart, Lung, and Blood Institute and National Institute for Health and Care Excellence guidelines recommend opioids for treating VOC-related pain.^33^,^34^ Due to the opioid crisis, the Centers for Disease Control and Prevention (CDC) published prescribing guidelines for chronic opioid use,^35^ and one study indicated the negative impact of these guidelines on SCD patients’ access to opioid therapy.^36^ The CDC later issued a letter clarifying that its guidelines should not negatively impact patients who suffer from pain conditions such as SCD,^37^ and the Centers for Medicare & Medicaid Services suggested excluding SCD patients from efforts to restrict opioid access.^38^

Little is known regarding the relationship between VOC events and opioid use. With the high prevalence of VOC events among SCD patients and the focus on opioid use nationally, there is a need to better understand this relationship. The objectives of this study were to examine the association between VOC events and outpatient opioid utilization and investigate the relationship between opioid prescriptions and health care resource utilization in patients with SCD. As SCD treatment options enter the market, understanding more about this relationship may help inform the use of new and innovative treatment strategies.

## METHODS

### Study Design and Data Source

This was a retrospective, observational cohort study using the Texas Medicaid administrative claims database. Data were retrieved from the Texas Health and Human Services Commission Medicaid inpatient, outpatient, prescription, and eligibility data files. Medical claims contained diagnoses codes (*International Classification of Diseases, Ninth Revision, Clinical Modification [ICD-9-CM] and ICD-10* codes) for inpatient and outpatient visits, current procedural terminology codes for visits and procedures, and relevant costs paid by Medicaid. Prescription claims included National Drug Code, dose, quantity, dispense date, days’ supply, and amount paid by Medicaid. Eligibility files contained demographic information including gender, age, race/ ethnicity, and dual eligibility information. The study was approved by The University of Texas at Austin Institutional Review Board.

### Study Sample

The study period ranged from September 1, 2011 until August 31, 2016. The index date was defined as the first date of SCD diagnosis present in the dataset, and the follow-up period was 12 months. Thus, the patient identification period was September 1, 2011 until August 31, 2015. Study subjects were included if they had at least one inpatient or two outpatient SCD diagnoses (*ICD-9-CM*=282.41, 282.42, 282.6, 282.60–282.69, *ICD-10*=D57, D57.x [except for D57.3; sickle cell trait], D57.xx) during the identification period; were continuously enrolled in Texas Medicaid during the 12-month postindex period; and were between 2 and 63 years of age at index date. Those who were 65 years of age or older at any time during the study were excluded due to their Medicaid–Medicare dual eligibility.

### Study Variables

The primary outcome was the mean number of opioid prescriptions filled during the 12-month study period. We examined the number of opioid prescriptions rather than the morphine milligram equivalent in order to better describe the volume of opioid prescribing for SCD patients. Secondary outcomes were all-cause and SCD-related health care resource utilization, including the number of hospitalizations, ED visits, outpatient visits, and prescriptions filled.

The primary independent variable was the mean number of VOC events. A VOC event was identified using *ICD-9-CM* codes (282.62, 282.64, 282.69, and 282.42) and *ICD-10* codes (D57.41, D57.419, D57.21, D57.219, D.57.0, D.57.00, D57.81, and D57.819). To avoid duplicate counting of the VOC events, VOC diagnoses that occurred within 7 days were not considered separate events. The secondary independent variable was the number of opioid prescriptions filled during the 12-month study period. Covariates for the multivariable analyses included demographics (age and gender) and clinical characteristics (number of nonopioid pain medications used; number of nonstudy SCD-related medications used [penicillin, folic acid, decitabine]; evidence of blood transfusion; number of SCD-related complications; number of SCD-related comorbid conditions; and Charlson Comorbidity Index^39^–^41^). Due to a large proportion of subjects with missing race/ethnicity information (20.2%), this variable was not included in the multivariable analyses.

### Statistical Analyses

Descriptive analyses were performed for all variables. Frequencies and percentages were presented for categorical variables; means and standard deviations (SD) were computed for continuous and count variables. Regarding multivariable analyses, negative binomial regression was employed for both study objectives, and all of the covariates listed previously were controlled. Incidence rate ratio (IRR), with its 95% CI and *P* value, was computed and is presented for each analysis.

## RESULTS

### Baseline Characteristics of Study Subjects

A total of 3368 patients with SCD who met the inclusion criteria were included in the study. Mean age of the patients was 21.4 (SD=14.9) years and most were ages 2–12 (36.6%) and 25–34 (16.5%). Regarding gender, there were slightly more females (54.4%) than males (45.6%). As expected, the majority of subjects with recorded race/ ethnicity were African American (2386/2687; 88.8%). (As previously mentioned, this variable was not included in multivariable analyses due to it being missing.) About 40% of subjects were prescribed penicillin (41.5%) and/or folic acid (37.8%) during the study period, whereas none of the subjects had claims for decitabine. Only 6.1% received a blood transfusion (Table 1). Almost 80% (79.3%) had SCD-related complications, with an average of 2.5 (SD=2.5), while 29.4% had at least one SCD-related comorbidity and 50.3% had a Charlson Comorbidity Index score of at least 1. The list of SCD-related complications and comorbidities can be found in Supplementary Material Tables S1 and S2, respectively.

### Association Between VOC Events and Opioid Prescriptions

During the 12-month postindex period, more than 60% (61.5%) of patients had at least one VOC event and, on average, there were 2.9 (SD=4.4) events per subject. Most patients experienced one (18.0%) or two (10.8%) events, but 8.6% had more than 10 VOC events during the 12-month follow-up period (Table 2).

More than 60% (65.8%) of patients received some type of prescription pain medication (opioid or nonopioid) with an average of 5.2 (SD=7.9) prescriptions per subject. More than half (58.7%) received opioids with an average of 4.2 (SD=7.2) prescriptions, and most (35.9%) received one to five opioid prescriptions during the study period. Opioids prescribed to the patients included codeine, fentanyl, hydrocodone, hydromorphone, methadone, morphine, oxycodone, and tramadol. Less than 40% (36.9%) received prescriptions for nonopioid pain medication with a mean of 1.1 (SD=2.1) prescriptions, and most of these prescriptions were for nonsteroidal anti-inflammatory drugs (Table 3).

Negative binomial regression showed that for every increase in VOC events, the number of opioid prescriptions was expected to increase by 9.5% (IRR=1.095; 95% CI: 1.078–1.111; *P* < 0.0001), while controlling for other covariates (Table 4).

### Association Between Opioid Prescriptions and Health Care Utilization

Table 5 presents all-cause and SCD-related health care utilization by SCD patients during the 12-month study period. The majority of hospitalizations (87.4%) and ED visits (70.7%) were SCD-related.

Results of negative binomial regression analyses examining the association between the number of opioid prescriptions and health care utilization among SCD patients are summarized in Table 6. Every increase in the number of opioid prescription claims was associated with an increase in the number of all-cause and SCD-related hospitalizations (by 2.3% and 2.9%, respectively), ED visits (by 3.7% and 4.5%, respectively), outpatient visits (by 0.6% and 1.1%, respectively), and prescriptions (by 6.7% and 4.0%, respectively). The association of other covariates with health care resource use can be found in Supplementary Material Tables S3–S6.

## DISCUSSION

To the authors’ knowledge, this was the first study to examine and quantify the association between the number of VOC events and the number of opioid prescriptions among SCD patients. As new treatment options for preventing VOC events are emerging, this study may provide a benchmark for how effective they may be in reducing opioid use.

During the 12-month study period, 61.5% of patients experienced at least one VOC event with an average of 2.9 (SD=4.4). In Shah et al.’s study using Medicaid Analytic eXtracts data from 2009 to 2013, 25.5% of all (adult and pediatric) SCD patients had one or more VOC events during one year.^42^ Their lower proportion can most likely be explained by the stricter VOC event definition they used. Shah et al. identified a VOC event when it required an inpatient stay, whereas this study included VOC events that were treated in the ED as well.

Regarding opioid utilization, this study found that 58.7% of patients had at least one opioid prescription during the study period. This finding is within the range of results reported by previous studies. Han et al. used 2009–2014 MarketScan Commercial and Medicaid data and found that 40% of SCD patients were prescribed opioids.^43^ Using the same database and the same years, Ballas et al. found that between 54% and 57% of commercial patients, and between 65% and 70% of Medicaid patients used opioids.^44^ In another study that analyzed medication-prescribing records at a comprehensive sickle cell center, Han et al. found that 75% of the patients had one or more opioid prescriptions.^45^ The mean number of opioid prescriptions per year (4.2, SD=7.2) in this study was lower than the finding from Medicaid SCD patients in Ballas et al. (9.0, SD=13.4).^44^ Our results for SCD patients were higher than the annual means reported for Medicaid patients in general who have received opioid prescriptions: 3.0 and 2.5 (SD not reported) prescriptions per enrollee in the Texas and Georgia Medicaid programs, respectively.^46^,^47^

Multivariable analyses showed that, for every increase in the number of VOC events, the number of opioid prescriptions was expected to increase by 9.5%. In other words, by preventing one VOC event, prescription opioid use for SCD patients could be reduced by almost 10%. In the secondary analyses, we found that decreased opioid use was associated with decreased all-cause and SCD-related health care resource utilization.

Therefore, for better management of SCD, emphasis should be given to preventing VOC events rather than restricting opioid use. Although opioids are the guideline-recommended therapy for SCD pain management,^33^,^34^,^48^ current efforts to curb opioid prescribing in the US have negatively impacted SCD patients who need these medications.^36^ Considering that pain crises occur throughout the lifespan of SCD patients, and inadequate pain management may lead to chronic and/or neuropathic pain requiring more extensive health care resource use, appropriate and adequate pain management is vital for this population.

To prevent VOC events, patients should be encouraged to take appropriate medications that are available in market, such as hydroxyurea, L-glutamine, crizanlizumab, and/or voxelotor, when they meet the criteria suggested by clinical guidelines.^33^,^49^,^50^ Hydroxyurea has shown its beneficial effects in reducing the number of VOC events for the last two decades.^19^,^24^,^25^,^51^ However, hydroxyurea prescribing is infrequent and even more infrequent than opioid prescribing among adults with SCD,^52^ which could be due to physicians’ concerns about blood-monitoring requirements and patients’ general nonadherence with the therapy.^53^,^54^ L-glutamine oral powder, approved by the US Food and Drug Administration in July 2017, can be added to hydroxyurea to further reduce VOC events. In addition, access to SCD specialists for adults with SCD needs to be improved. Due to the lack of specialists, adult SCD patients visit the ED more often and visit hematology/oncology specialists less often than do children.^52^

When an SCD pain crisis does occur, it should be treated appropriately and adequately. As found in the CDC’s clarification letter responding to concerns issued by the American Society of Clinical Oncology, the American Society of Hematology, and the National Comprehensive Cancer Network, special consideration should be given to SCD patients’ pain management, and access to appropriate pain management should not be limited.^37^ In addition, providers and payers should be educated regarding the National Heart, Lung, and Blood Institute’s guidelines for the management of SCD and the importance of treating pain in this population.

### Limitations

This study had several limitations that should be noted. First, because this was a retrospective study using claims data, the research scope was necessarily limited by the information provided in the database. Potential confounders, such as fetal hemoglobin level and VOC events treated at home, could not be identified. Validity of the recorded diagnoses and procedure codes depended on coding accuracy. Second, opioid use may not have been SCD-related. Patients may have used opioids to treat pain not associated with their SCD. Additionally, we examined the number of opioid prescriptions and not morphine milligram equivalent doses and compared our results with other studies that reported numbers of opioid prescriptions.^44^,^46^,^47^ Our aim was to assess how many prescriptions were received for pain management rather than the intensity of pain management, which could be difficult to interpret when comparing adults to adolescents and children. Third, since all the study variables were measured during the 12-month follow-up period, causality could not be established. Fourth, generalization of the study results may not be valid beyond our study population. Findings of this study should be interpreted with consideration for Texas Medicaid program specifics, including patients, geographic location, and program policies.

## CONCLUSIONS

The majority of SCD patients experience pain crises that require appropriate management, including prescription opioids. We found that every increase in the number of pain crises is associated with a 9.5% increase in opioid prescriptions among this population. Increased opioid prescription was further associated with higher health care resource utilization. Health care providers and payers should focus more on the prevention of pain crises, rather than restricting opioid prescribing, for better disease management of SCD.

## Supplementary Information



## Figures and Tables

**Table 1:** Baseline Characteristics for Patients with SCD (N = 3368)

	SCD Patients (N = 3368)	
	N	%
**Demographic Characteristics**		
Age (Mean±SD, Year)	21.4 ± 14.9	
Age Groups		
2–12	1231	36.6
13–17	407	12.1
18–24	524	15.6
25–34	555	16.5
35–44	335	10.0
45–54	234	7.0
55–63	82	2.4
Gender		
Female	1832	54.4
Male	1536	45.6
Race/Ethnicity		
African American	2386	70.8
Caucasian	97	2.9
Hispanic	181	5.4
Asian	23	0.7
Others/unknown	681	20.2
**Clinical Characteristics**		
Nonstudy SCD-Related Medication Use		
Medication		
Penicillin	1399	41.5
Folic acid	1272	37.8
Total number of nonstudy SCD medications		
None	1354	40.2
1 (either penicillin or folic acid)	1357	40.3
2 (both penicillin and folic acid)	657	19.5
Blood Transfusion		
Yes	204	6.1
No	3164	93.9
SCD-Related Complications^a^		
Mean±SD	2.5 ± 2.5	
Yes	2669	79.3
No	699	20.8
SCD-Related Comorbidities^a^		
Mean±SD	0.5 ± 1.0	
0	2377	70.6
1	549	16.3
2	225	6.7
3+	217	6.4
Charlson Comorbidity Index Scores		
Mean±SD	1.1 ± 1.8	
0	1675	49.7
1	913	27.1
2+	780	23.2

Abbreviation: SCD, sickle cell disease; SD, standard deviation.

a The list of SCD-related complications and comorbidities can be found in Supplementary Material Tables S1 and S2, respectively.

**Table 2:** VOC Events among Patients with SCD (N = 3368)

VOC Events	SCD Patients (N = 3368)	
	N	%
Mean±SD	2.9 ± 4.4	
0	1297	38.5
1	605	18.0
2	362	10.8
3	249	7.4
4	134	4.0
5	131	3.9
6	101	3.0
7	74	2.2
8	69	2.1
9	57	1.7
10+	289	8.6

Abbreviations: SCD, sickle cell disease; VOC, vaso-occlusive crisis; SD, standard deviation.

**Table 3:** Pain Medication Use among Patients with SCD (N = 3368)

Pain Medication Use	SCD Patients (N = 3368)	
	N	%
**Opioid and Nonopioid pain medication**		
Pain medication use (all): mean±SD, claims	5.2 ± 7.9	
Yes	2217	65.8
No	1151	34.2
**Opioid Medication**		
Opioid use: mean±SD, claims	4.2 ± 7.2	
Yes	1978	58.7
No	1390	41.3
1–5	1210	35.9
6–10	313	9.3
11–15	191	5.7
16–20	112	3.3
>20	152	4.5
**Nonopioid Medication**		
Nonopioid pain medication use: mean±SD, claims	1.1 ± 2.1	
Yes	1243	36.9
No	2125	63.1
Nonsteroidal anti-inflammatory drugs	1173	34.8
Acetaminophen	136	4.0
Others (butalbital + acetaminophen + caffeine)	42	1.3

Abbreviations: SCD, sickle cell disease; VOC, vaso-occlusive crisis; SD, standard deviation.

**Table 4:** Results of Negative Binomial Regression Examining the Association Between the Number of VOCs and the Number of Opioid Prescriptions Prescribed to Patients with SCD (N = 3368)

	IRR	95% Confidence Limits		*P* value

		Lower	Upper	
Number of VOC events	1.095	1.078	1.111	<0.0001

Abbreviations: IRR, incidence rate ratio; SCD, sickle cell disease; VOC, vaso-occlusive crisis.

Other significant covariates with higher expected opioid use included: age groups (13 and older compared to 2–12), increase in number of nonopioid pain medications, increase in number of nonstudy SCD-related medications, and increase in number of SCD-related complications.

**Table 5:** All-Cause and Disease-Related Health Care Utilization among Patients with SCD (N = 3368)

	All-Cause		SCD-Related	
	Mean	SD	Mean	SD
Hospitalization	1.6	2.7	1.4	2.6
ED visits	4.8	10.1	3.4	8.6
Outpatient visits	24.1	28.0	10.0	13.3
Prescription claims filled	23.6	28.8	4.7	7.0

Abbreviations: ED, emergency department; SCD, sickle cell disease; SD, standard deviation.

**Table 6:** Results of Negative Binomial Regression Analyses Examining the Association Between the Number of Opioid Prescriptions and Health Care Utilization among Patients with SCD (N = 3368)

Health Care Utilization	IRR	95% Confidence Limits		*P* value

		Lower	Upper	
Hospitalizations (All-cause)	1.023	1.017	1.029	<0.0001
Hospitalizations (SCD-related)	1.029	1.023	1.036	<0.0001
ED visits (All-cause)	1.037	1.031	1.043	<0.0001
ED visits (SCD-related)	1.045	1.038	1.053	<0.0001
Outpatient visits (All-cause)	1.006	1.002	1.009	0.0037
Outpatient visits (SCD-related)	1.011	1.006	1.015	<0.0001
Prescription utilization (All-cause)	1.067	1.062	1.073	<0.0001
Prescription utilization (SCD-related)	1.040	1.034	1.046	<0.0001

Abbreviations: ED, emergency department; IRR, incidence rate ratio; SCD, sickle cell disease.
