# Supplementary material for: Association between Vaso-occlusive Crises and Opioid Prescriptions among Patients with Sickle Cell Disease: A Retrospective Claims-based Study
Source: J Health Econ Outcomes Res. 2020 Jun 26;7(1):94–101. doi: 10.36469/jheor.2020.13348 (PMC7343345; doi:10.36469/jheor.2020.13348)
Supplement: Supplementary file 1 [file jheor-7-1-13348-s01.pdf]

JHEOR Journal of Health Economics and Outcomes Research

Supplementary Online Material

Kang HA, Barner JC, Richards KM, Bhor M, Paulose J, Kutlar A. Association between vaso-occlusive crises and opioid prescriptions among patients with sickle cell disease: A retrospective claims-based study. *JHEOR*. 2020;7(1):95-101.
doi: 10.36469/jheor.2020.13348

Table S1. ICD-9-CM and ICD-10 Codes for Complications of SCD
Table S2. ICD-9-CM and ICD-10 Codes for Comorbidities Associated with SCD
Table S3. Negative Binomial Regression Examining the Relationship Between Opioid Use and All-Cause and SCD-Related Hospitalizations (N = 3368)
Table S4. Negative Binomial Regression Examining the Relationship Between Opioid Use and All-Cause and SCD-Related ED Visits (N = 3368)
Table S5. Negative Binomial Regression Examining the Relationship Between Opioid Use and All-Cause and SCD-Related Outpatient Visits (N = 3368)
Table S6. Negative Binomial Regression Examining the Relationship Between Opioid Use and All-Cause and SCD-Related Prescriptions (n = 3368)

This supplementary material has been provided by the authors to give readers additional information about their work.

This is an open-access article distributed under the terms of the Creative Commons Attribution 4.0 International License (CCBY-4.0). View this license's legal deed at http://creativecommons.org/licenses/by/4.0 and legal code at http://creativecommons.org/licenses/by/4.0/legalcode for more information.

Table S1. *ICD-9-CM* and *ICD-10* Codes for Complications of SCD

Anatomy	Disease/Disorder	*ICD-9-CM*	*ICD-10*
Cardiovascular	CHF	428.0	I50.2, I50.2x, I50.3, I50.3x, I50.4, I50.4x, I50.9
	Cardiomegaly	429.3	I51.7
	Cardiomyopathy	425.9	I42, I42x (except I42.6; alcoholic cardiomyopathy, I42.7; cardiomyopathy due to drug and external agent)
	Diastolic dysfunction	429.9 (heart disease, unspecified)	I51.9 (heart disease, unspecified)
	Dysrhythmia	427.9	I49.9
	Stroke,[a] specified as follows:		
	Ischemic	433.01, 433.11, 433.21, 433.31, 433.81, 433.91, 434.01, 434.11, 434.91	I63, I63.x, I63.xx
	Hemorrhagic	430, 431	I60, I60.x, I60.xx, I61, I61.x
	TIA	435, 435.x	G45, G45.x
Hepatic	Gallstones, cholelithiasis[a]	574, 574.x, 674.xx	K80.x, K80.xx
	Cholecystitis[a]	575.0, 575.1, 575.10, 575.11, 575.12	K81.x
	Bilirubinemia[a]	277.4	R82.2
	Biliary sludge[a]	576.8 (other specified disorders of biliary tract)	K83.8 (other specified diseases of biliary tract)
	Acute hepatic sequestration[a]	573.8 (other specified disorders of liver)	K76.89 (other specified diseases of liver)
	Acute intrahepatic cholestasis[a]	576.2 (obstruction of bile duct)	K83.1 (obstruction of bile duct)
	Other gall bladder disease[b,c]	575, 575.x, 575.xx	K82, K82.x, K82.xx
Renal	Hematuria	599.7, 599.7x	R31, R31.x, R31.xx
	Proteinuria including microalbuminuria	791.0	R80. R80.x
	Polyuria	788.42	R35, R35.x
	Nephrolithiasis	592	N20.0
	Papillary necrosis	583.7 (nephritis and nephropathy, not specified as acute or chronic, with lesion of renal medullary necrosis)	N17.2
	CKD	593.9 (unspecified disorder of kidney and ureter)	N18.9 (CKD, stages I–V, to be more specific)
	Glomerulonephritis (acute, chronic)	580, 580.x, 580.xx, 582, 582.x, 582.xx	N00.2-5, N00.7, N01.2-5, N01.7, N02.2-5, N02.7, N03.2-5, N03.7, N04.2-5, N04.7, N05.2-5, N05.7, N06.2-5, N06.7, N07.2-5, N07.7
	Renal failure, acute or chronic[c]	584, 584.x, 585, 585.x 586	N17, N17.x, N18, N18.x, N19
Hematology	Thrombosis[a]	452, 453, 453.x, 453.xx	I81, I82.x, I82.xx, I82.xxx
	Aplastic anemia[a]	284.89	D61.89
	Myelosuppression	289.9 (unspecified diseases of blood and blood-forming organs)	D75.9 (disease of blood and blood-forming organs, unspecified)
	Hemolytic anemia[b]/hyperhemolysis[a] (delayed hemolytic transfusion reaction)	283.1, 283.10, 283.19, 283.9	D59.4, D59.8, D59.9
Spleen	Splenic sequestration[a]	289.52	D57.02 (Hb-SS disease with splenic sequestration), D57.212 (sickle-cell/Hb-C disease with splenic sequestration), D57.412 (sickle-cell thalassemia with splenic sequestration)
	Splenic infarcts	289.59 (other diseases of spleen)	D73.5
	Hypersplenism	289.4	D73.1
Genitourinary	Priapism[a,b,c]	607.3	N48.3, N48.30, N48.32, N48.39

Table S1. *ICD-9-CM* and *ICD-10* Codes for Complications of SCD (Continued)

Anatomy	Disease/Disorder	*ICD-9-CM*	*ICD-10*
Pulmonary	Pneumococcal pneumonia	481	J13
	Pulmonary embolism[a]	415.1, 415.19, 416.2	I26.09, I26.99, I27.82
	Pulmonary hypertension[b]	416.8	I27.2, I27.21, I29.29, I27.8, I27.89, I27.9
	ACS[a]	517.3	D57.01, D57.211, D57.411, D57.811
	Asthma	493.1, 493.1x, 493.2, 493.2x, 493.9, 493.9x	J45.2, J45.2x, J45.3, J45.3x, J45.4, J45.4x, J45.5, J45.5x, J45.90, J45.90x
	Upper respiratory tract infections	465, 465.x	J00, J01, J01.x, J01.xx, J02, J02.x, J03, J03.x, J03.xx, J04, J04.x, J04.xx, J05, J05.x, J05.xx, J06, J06.x
Neurology	Acute sickle cell pain episode / VOC crisis[a]	282.62, 282.64, 282.69, 282.42	D57.0, D57.00, D57.21, D57.219, D57.81, D57.819, D57.41, D57.419
	Chronic pain syndrome[b] (neuropathic pain)	729.2	M54.1, M54.1x, M79.2
	Seizures	345, 345.x, 345.xx, 780.3	G40, G40.x, G40.xx, R56.9
	Moyamoya	437.5	I67.5
	Obstructive sleep apnea	327.23	G47.33
Ocular	Orbital cellulitis	376.01	H05.01, H05.01x
	Retinopathy[b,c]	362.72	H36 (retinal disorders in diseases classified elsewhere)
	Retinal vascular occlusion (orbital infarction)	362.3, 362.3x	H34 (retinal vascular occlusions), H34.x, H34.xx, H43.xxx
	Retinal detachment	361.2	H33.2
	Hyphema	364.41	H21.0, H21.0x
Musculoskeletal	Avascular necrosis (bone infarction; osteonecrosis)[a,b,c]	733.4x	M87.3, M87.3x, M87.3xx, M87.8, M87.8x, M87.8xx, M87.9
	Osteomyelitis	730, 730.x, 730.xx	M86, M86.x, M86.xx, M86.xxx
Dermatology	Hand foot syndrome (dactylitis)	729.5	M79.644, M79.645, M79.646 (pain in fingers)
	Leg ulcers[b,c]	454.0, 454.2, 707.1, 707.1x	I83.0, I83.0x, I83.0xx, I83.2, I83.2x, I83.2xx, L97, L97.x, L97.xx, L97.xxx
	Cellulitis	681, 681.x, 681.xx, 682, 682.x	L03.x, L03.xx, L03.xxx
Gastrointestinal	Constipation	564.0, 564.0x	K59.0. K59.0x
Other infectious and parasitic diseases	Meningitis[a]	047, 047.x, 320, 320.x, 320.xx, 321, 321.x, 322, 322.x	A87, A87.x, G00, G00.x, G01, G02, G03, G03.x
	Sepsis/Septicemia[a]	995.91, 995.92, 038, 038.x, 038.xx	A41, A41.x, A41.xx, R65.2, R65.2x
Others	Hypovolemia	276.52	E86.1
	Iron overload[b]	275.02	E83.111
	Infectious and noninfectious systemic inflammation with acute organ dysfunction (multiorgan failure)	995.92, 995.94	R65.11, R65.20, R65.21
	Neoplasms benign and malignant cancer	140–239	C00–D49

Abbreviations: ACS, acute chest syndrome; CHF, congestive heart failure; CKD, chronic kidney disease; Hb, hemoglobin; ICD-CM, International Classification of Diseases Clinical Modification; SCD, sickle cell disease; TIA, transient ischemic attack; VOC, vaso-occlusive crisis.
[a] Acute.
[b] Chronic.
[c] Organ damage.

Table S2. ***ICD-9-CM*** **and** ***ICD-10*** **Codes for Comorbidities Associated with SCD**

Description	*ICD-9-CM*	*ICD-10*
Cardiovascular disease		
CHF	428.0	I50.2, I50.3, I50.4, I50.9
CAD	414.00	I25.10
Valvular heart disease	394.x, 395.x, 396.x	I34.x, I35.x, I36.x, I37.x, I38.x, I39.x
Diabetes Mellitus	250.x	E11.x
Hyperlipidemia	272.0x, 272.1x, 272.2x, 272.4x,	E78.0x, E78.1x, E78.2x, E78.4x, E78.5x
Hypertension	401.x	I10
Osteoporosis	733.0x	M80.x, M81.x
Gout	274.x	M10.x
Obesity	278, 278.0x	E66.x

Abbreviations: CAD, coronary artery disease; CHF, congestive heart failure; SCD, sickle cell disease.

Table S3. Negative Binomial Regression Examining the Relationship Between Opioid Use and All-Cause and SCD-Related Hospitalizations (N = 3368)

Outcome: All-Cause Hospitalization	Incidence Rate Ratio	95% Confidence Limits		Wald X^2	*P*-value
		Lower	Upper		
**Number of Opioids (claims)**	**1.023**	**1.017**	**1.029**	**60.66**	**<0.0001**
13–17[a]	1.237	1.050	1.457	6.44	0.0111
18–24[a]	2.497	2.173	2.869	166.46	<0.0001
25–34[a]	2.366	2.055	2.725	142.82	<0.0001
35–44[a]	1.600	1.348	1.900	28.81	<0.0001
45 and older[a]	1.213	1.002	1.469	3.94	0.0471
Male[a]	0.985	0.903	1.075	0.12	0.7313
Number of non-opioid pain medications	1.005	0.984	1.026	0.20	0.6532
Number of non-study SCD-related medications[b]	1.009	0.999	1.018	3.19	0.0742
Evidence of transfusion[a]	1.052	0.890	1.242	0.35	0.5543
Number of SCD-related complications	1.294	1.269	1.320	652.10	<0.0001
Number of SCD-related comorbidities	0.997	0.949	1.047	0.02	0.8928
CCI≥1[a]	1.128	1.022	1.246	5.68	0.0172
**Outcome: SCD-Related Hospitalization**	**Incidence Rate Ratio**	**95% Confidence Limits**		**Wald X^2**	***P*-value**
		**Lower**	**Upper**		
**Number of Opioids (claims)**	**1.029**	**1.023**	**1.036**	**78.09**	**<0.0001**
13–17[a]	1.232	1.032	1.472	5.30	0.0213
18–24[a]	2.501	2.148	2.913	139.28	<0.0001
25–34[a]	2.357	2.017	2.754	116.56	<0.0001
35–44[a]	1.516	1.253	1.835	18.30	<0.0001
45 and older[a]	1.123	0.907	1.391	1.13	0.2875
Male[a]	1.042	0.946	1.146	0.69	0.4051
Number of non-opioid pain medications	0.998	0.975	1.022	0.03	0.8596
Number of non-study SCD-related medications[b]	1.013	1.003	1.023	6.24	0.0125
Evidence of transfusion[a]	1.091	0.907	1.312	0.86	0.3547
Number of SCD-related complications	1.333	1.303	1.364	621.40	<0.0001
Number of SCD-related comorbidities	0.903	0.854	0.956	12.38	0.0004
CCI≥1[a]	1.087	0.975	1.212	2.25	0.1333

Abbreviations: CCI, Charlson Comorbidity Index; SCD, sickle cell disease.
[a] Reference categories: age 2-12; Female; No evidence of transfusion; CCI=0.
[b] Penicillin and folic acid.

Table S4. Negative Binomial Regression Examining the Relationship Between Opioid Use and All-Cause and SCD-Related ED Visits (N = 3368)

Outcome: All-Cause ED Visits	Incidence Rate Ratio	95% Confidence Limits		Wald X^2	*P*-value
		Lower	Upper		
**Number of Opioids (claims)**	**1.037**	**1.031**	**1.043**	**156.90**	**<0.0001**
13–17[a]	0.971	0.844	1.118	0.16	0.6852
18–24[a]	2.281	2.016	2.581	171.37	<0.0001
25–34[a]	2.696	2.382	3.052	246.27	<0.0001
35–44[a]	1.833	1.578	2.131	62.54	<0.0001
45 and older[a]	1.224	1.039	1.442	5.86	0.0155
Male[a]	0.971	0.899	1.049	0.55	0.4586
Number of non-opioid pain medications	1.003	0.985	1.022	0.13	0.7189
Number of non-study SCD-related medications[b]	0.995	0.987	1.003	1.37	0.2411
Evidence of transfusion[a]	0.777	0.664	0.909	9.94	0.0016
Number of SCD-related complications	1.246	1.223	1.269	542.08	<0.0001
Number of SCD-related comorbidities	1.057	1.009	1.108	5.39	0.0203
CCI≥1[a]	1.134	1.039	1.237	8.00	0.0047
**Outcome: SCD-Related ED Visits**	**Incidence Rate Ratio**	**95% Confidence Limits**		**Wald X^2**	***P*-value**
		**Lower**	**Upper**		
**Number of Opioids (claims)**	**1.045**	**1.038**	**1.053**	**153.70**	**<0.0001**
13–17[a]	1.009	0.851	1.196	0.01	0.9164
18–24[a]	2.329	2.005	2.706	121.99	<0.0001
25–34[a]	2.662	2.288	3.097	160.61	<0.0001
35–44[a]	1.820	1.517	2.185	41.42	<0.0001
45 and older[a]	1.011	0.826	1.239	0.01	0.9132
Male[a]	1.097	0.999	1.204	3.78	0.0518
Number of non-opioid pain medications	0.983	0.961	1.006	2.07	0.1504
Number of non-study SCD-related medications[b]	0.998	0.988	1.007	0.25	0.6149
Evidence of transfusion[a]	0.835	0.690	1.010	3.47	0.0627
Number of SCD-related complications	1.321	1.291	1.352	557.15	<0.0001
Number of SCD-related comorbidities	0.914	0.862	0.969	9.08	0.0026
CCI≥1[a]	1.035	0.931	1.151	0.41	0.5234

Abbreviations: CCI, Charlson Comorbidity Index; ED, emergency department; SCD, sickle cell disease.
[a] Reference categories: age 2–12; Female; No evidence of transfusion; CCI=0.
[b] Penicillin and folic acid.

Table S5. Negative Binomial Regression Examining the Relationship Between Opioid Use and All-Cause and SCD-Related Outpatient Visits (N = 3368)

Outcome: All-Cause Outpatient Visits	Incidence Rate Ratio	95% Confidence Limits		Wald X^2	*P*-value
		Lower	Upper		
**Number of Opioids (claims)**	**1.006**	**1.002**	**1.009**	**8.45**	**0.0037**
13–17[a]	0.917	0.838	1.003	3.60	0.0579
18–24[a]	1.010	0.928	1.099	0.05	0.8190
25–34[a]	1.154	1.058	1.258	10.40	0.0013
35–44[a]	1.177	1.060	1.307	9.31	0.0023
45 and older[a]	1.147	1.024	1.284	5.61	0.0178
Male[a]	0.941	0.892	0.993	4.87	0.0273
Number of non-opioid pain medications	1.008	0.995	1.021	1.61	0.2051
Number of non-study SCD-related medications[b]	1.020	1.015	1.026	54.99	<0.0001
Evidence of transfusion[a]	1.575	1.414	1.756	67.59	<0.0001
Number of SCD-related complications	1.093	1.079	1.107	184.98	<0.0001
Number of SCD-related comorbidities	1.204	1.164	1.245	116.49	<0.0001
CCI≥1[a]	1.400	1.317	1.488	115.63	<0.0001
**Outcome: SCD-Related Outpatient Visits**	**Incidence Rate Ratio**	**95% Confidence Limits**		**Wald X^2**	***P*-value**
		**Lower**	**Upper**		
**Number of Opioids (claims)**	**1.011**	**1.006**	**1.015**	**21.88**	**<0.0001**
13–17[a]	0.928	0.837	1.030	1.97	0.1600
18–24[a]	1.031	0.935	1.138	0.38	0.5367
25–34[a]	1.261	1.141	1.393	20.76	<0.0001
35–44[a]	1.176	1.045	1.324	7.25	0.0071
45 and older[a]	1.013	0.891	1.151	0.04	0.8489
Male[a]	0.954	0.898	1.014	2.26	0.1331
Number of non-opioid pain medications	0.998	0.983	1.012	0.09	0.7648
Number of non-study SCD-related medications[b]	1.030	1.024	1.037	91.28	<0.0001
Evidence of transfusion[a]	1.832	1.621	2.070	94.10	<0.0001
Number of SCD-related complications	1.148	1.131	1.166	333.61	<0.0001
Number of SCD-related comorbidities	0.983	0.945	1.022	0.72	0.3963
CCI≥1[a]	1.293	1.205	1.387	51.25	<0.0001

Abbreviations: CCI, Charlson Comorbidity Index; SCD, sickle cell disease.
[a] Reference categories: age 2–12; Female; No evidence of transfusion; CCI=0.
[b] Penicillin and folic acid.

Table S6. Negative Binomial Regression Examining the Relationship Between Opioid Use and All-Cause and SCD-Related Prescriptions (n = 3368)

Outcome: All-Cause Prescriptions	Incidence Rate Ratio	95% Confidence Limits		Wald X^2	*P*-value
		Lower	Upper		
**Number of Opioids (claims)**	**1.067**	**1.062**	**1.073**	**541.89**	**<0.0001**
13–17[a]	0.954	0.860	1.057	0.82	0.3657
18–24[a]	0.751	0.680	0.829	32.10	<0.0001
25–34[a]	0.712	0.643	0.788	42.89	<0.0001
35–44[a]	0.721	0.637	0.816	26.84	<0.0001
45 and older[a]	0.897	0.785	1.024	2.61	0.1063
Male[a]	0.900	0.845	0.958	10.96	0.0009
Number of non-opioid pain medications	1.103	1.086	1.121	146.89	<0.0001
Number of non-study SCD-related medications[b]	1.080	1.073	1.087	550.31	<0.0001
Evidence of transfusion[a]	0.987	0.868	1.122	0.04	0.8376
Number of SCD-related complications	1.011	0.996	1.026	2.15	0.1428
Number of SCD-related comorbidities	1.231	1.184	1.280	109.87	<0.0001
CCI≥1[a]	1.539	1.435	1.651	145.02	<0.0001
**Outcome: SCD-Related Prescriptions[c]**	**Incidence Rate Ratio**	**95% Confidence Limits**		**Wald X^2**	***P*-value**
		**Lower**	**Upper**		
**Number of Opioids (claims)**	**1.040**	**1.034**	**1.046**	**176.65**	**<0.0001**
13–18[a]	0.960	0.857	1.075	0.50	0.4774
18–24[a]	0.759	0.679	0.848	23.63	<0.0001
25–34[a]	0.681	0.606	0.764	42.37	<0.0001
35–44[a]	0.599	0.517	0.695	46.21	<0.0001
45 and older[a]	0.558	0.475	0.656	49.97	<0.0001
Male[a]	1.039	0.969	1.114	1.13	0.2870
Number of non-opioid pain medications	1.035	1.018	1.053	16.43	<0.0001
Number of non-study SCD-related medications[b]	1.205	1.195	1.214	2029.60	<0.0001
Evidence of transfusion[a]	1.071	0.926	1.238	0.85	0.3567
Number of SCD-related complications	1.037	1.020	1.055	18.17	<0.0001
Number of SCD-related comorbidities	0.909	0.868	0.952	16.34	<0.0001
CCI≥1[a]	1.004	0.927	1.088	0.01	0.9150

Abbreviations: CCI, Charlson Comorbidity Index; SCD, sickle cell disease.
[a] Reference categories: age 2–12; female; no evidence of transfusion; CCI=0.
[b] Penicillin and folic acid.
[c] Hydroxyurea, penicillin, and folic acid
